# Comparison of Radiomic Models Based on Low-Dose and Standard-Dose CT for Prediction of Adenocarcinomas and Benign Lesions in Solid Pulmonary Nodules

**DOI:** 10.3389/fonc.2020.634298

**Published:** 2021-02-02

**Authors:** Jieke Liu, Hao Xu, Haomiao Qing, Yong Li, Xi Yang, Changjiu He, Jing Ren, Peng Zhou

**Affiliations:** Department of Radiology, Sichuan Cancer Hospital & Institute, Sichuan Cancer Center, School of Medicine, University of Electronic Science and Technology of China, Chengdu, China

**Keywords:** radiomics, low-dose computed tomography, lung cancer screening, lung adenocarcinoma, benign lesion, solid pulmonary nodule

## Abstract

**Objectives:**

This study aimed to develop radiomic models based on low-dose CT (LDCT) and standard-dose CT to distinguish adenocarcinomas from benign lesions in patients with solid solitary pulmonary nodules and compare the performance among these radiomic models and Lung CT Screening Reporting and Data System (Lung-RADS). The reproducibility of radiomic features between LDCT and standard-dose CT were also evaluated.

**Methods:**

A total of 141 consecutive pathologically confirmed solid solitary pulmonary nodules were enrolled including 50 adenocarcinomas and 48 benign nodules in primary cohort and 22 adenocarcinomas and 21 benign nodules in validation cohort. LDCT and standard-dose CT scans were conducted using same acquisition parameters and reconstruction method except for radiation dose. All nodules were automatically segmented and 104 original radiomic features were extracted. The concordance correlation coefficient was used to quantify reproducibility of radiomic features between LDCT and standard-dose CT. Radiomic features were selected to build radiomic signature, and clinical characteristics and radiomic signature were combined to develop radiomic nomogram for LDCT and standard-dose CT, respectively. The performance of radiomic models and Lung-RADS was assessed by area under curve (AUC) of receiver operating characteristic curve, sensitivity, and specificity.

**Results:**

Shape and first order features, and neighboring gray tone difference matrix features were highly reproducible between LDCT and standard-dose CT. No significant differences of AUCs were found among radiomic signature and nomogram of LDCT and standard-dose CT in both primary and validation cohort (0.915 *vs.* 0.919 *vs.* 0.898 *vs.* 0.909 and 0.976 *vs.* 0.976 *vs.* 0.985 *vs.* 0.987, respectively). These radiomic models had higher specificity than Lung-RADS (all correct *P* < 0.05), while there were no significant differences of sensitivity between Lung-RADS and radiomic models.

**Conclusions:**

The diagnostic performance of LDCT-based radiomic models to differentiate adenocarcinomas from benign lesions in solid pulmonary nodules were equivalent to that of standard-dose CT. The LDCT-based radiomic model with higher specificity and lower false-positive rate than Lung-RADS might help reduce overdiagnosis and overtreatment of solid pulmonary nodules in lung cancer screening.

## Introduction

Lung cancer is the leading cause of cancer-related death worldwide (1–3). Low-dose computed tomography (LDCT) has been widely recommended for lung cancer screening as it can reduce the mortality (4, 5), but concerns about the high false-positive rate of diagnosis and the following overtreatment are also emerging (4, 6–8). Radiomics, *via* high-throughput extraction of features from imaging data, has been applied to risk prediction, diagnostic discrimination, and disease progression, and improves decision-making in oncology (9–11). In recent years, a large number of studies build radiomic models using either LDCT (12–17) or standard-dose CT data (18–20) to predict malignancy of solitary pulmonary nodules, however, one key question that remains unanswered is whether the performance of LDCT-based radiomic model and underlying significant features are equivalent to that of standard-dose CT.

Concurrent with the recent prosperities on radiomics, the effect of scan acquisition parameters on the reproducibility of quantitative radiomic features aroused concerns of researchers. Studies in phantom and *in vivo* demonstrated that scanner variability, radiation dose, reconstruction method, and slice thickness did affect the quantification of many radiomic features (21–25). Therefore, the LDCT-based radiomic model may be not identical to that of standard-dose CT due to radiation dose reduction even though the other acquisition parameters are consistent. To study the effect of radiation dose reduction on radiomic features *in vivo*, Lo et al. applied the noise addition methods to simulate dose reduction conditions (22), while Solomon et al. repeated scan with half standard dose (23). Their results indicated some texture features were not reproducible when reducing radiation dose. However, the reproducibility of radiomic features of solitary pulmonary nodules between LDCT for lung cancer screening and standard-dose CT examinations remains unaddressed.

Adenocarcinoma is the most prevalent histologic type of lung cancer (26, 27), making it the most common true-positive finding in lung cancer screening (4, 5). Granulomas often appear as spiculated or lobulated solid nodules and are fluorodeoxyglucose avid, and therefore mimic invasive adenocarcinomas, representing the most confounding false-positive findings in lung cancer screening (4, 28). Many investigators attempted to distinguish granulomas from adenocarcinomas using radiomic features (29–33), but none of them used low-dose acquisition parameters. Besides, the radiomic model without including non-specific inflammation, hamartoma, and other benign lesions might limit its utility in lung cancer screening.

Thus, the present study aimed to develop radiomic models based on LDCT and standard-dose CT from same subjects to distinguish adenocarcinomas from benign lesions in patients with solid solitary pulmonary nodules and compare the performance among these radiomic models and Lung CT Screening Reporting and Data System (Lung-RADS). We also assessed the reproducibility of radiomic features of solid solitary pulmonary nodules between LDCT and standard-dose CT examinations.

## Materials and Methods

### Pulmonary Nodules

This study was approved by the Institutional Review Board and the requirement for informed consent was waived as the data were analyzed retrospectively and anonymously.

A total of 141 solid solitary pulmonary nodules (72 adenocarcinomas and 69 benign nodules) were consecutively included in this study from April 2019 and May 2020, according to the following inclusion criteria: 1) detection of solid solitary pulmonary nodule without calcification for typical benign lesion; 2) LDCT obtained from lung cancer screening; 3) standard-dose CT obtained within 24 h after LDCT to evaluate hilar and mediastinal lymph nodes; 4) pathologically confirmed. The exclusion criteria were as follows: 1) history of cancer in previous 5 years; 2) images of poor quality with respiratory and movement artifacts; 3) nodules with undefined border resulting in poor segmentation.

We divided the nodules into two independent cohorts according to a ratio of 7:3 and the date of inclusion. Fifty adenocarcinomas and 48 benign nodules enrolled between April 2019 and November 2019 constituted the primary cohort, and 22 adenocarcinomas and 21 benign nodules enrolled between November 2019 and May 2020 constituted the validation cohort. The radiologist (HQ, with 7 years of experience in thoracic radiology) who was blinded to the final diagnosis performed categorization on nodules according to Lung-RADS (34).

### Image Acquisition

All LDCT and standard-dose CT scans were performed on a 256-slice multi-detector CT scanner (Brilliance iCT, Philips Healthcare, Amsterdam, Netherlands), using the following acquisition parameters: tube voltage of 100 kV and tube current of 20 or 30 mAs for LDCT, tube voltage of 120 kV and tube current of 100 to 250 mAs for standard-dose CT, standard resolution mode, detector collimation of 128 × 0.625 mm, helical pitch of 0.915, and gantry rotation time of 0.4 s. All the raw datasets were then reconstructed using the hybrid iterative reconstruction method (iDose4, level 6, Philips Healthcare, Amsterdam, Netherlands) with standard reconstruction filter for body, slice thickness of 0.625 mm, slice increment of 0.625 mm, field of view of 350 mm × 350 mm, and matrix of 1,024 × 1,024. The estimated effective dose of LDCT scan for all subjects was 0.68 ± 0.11 mSv (range from 0.40 to 0.93).

### Segmentation and Radiomic Features Extraction

**Figure 1:**
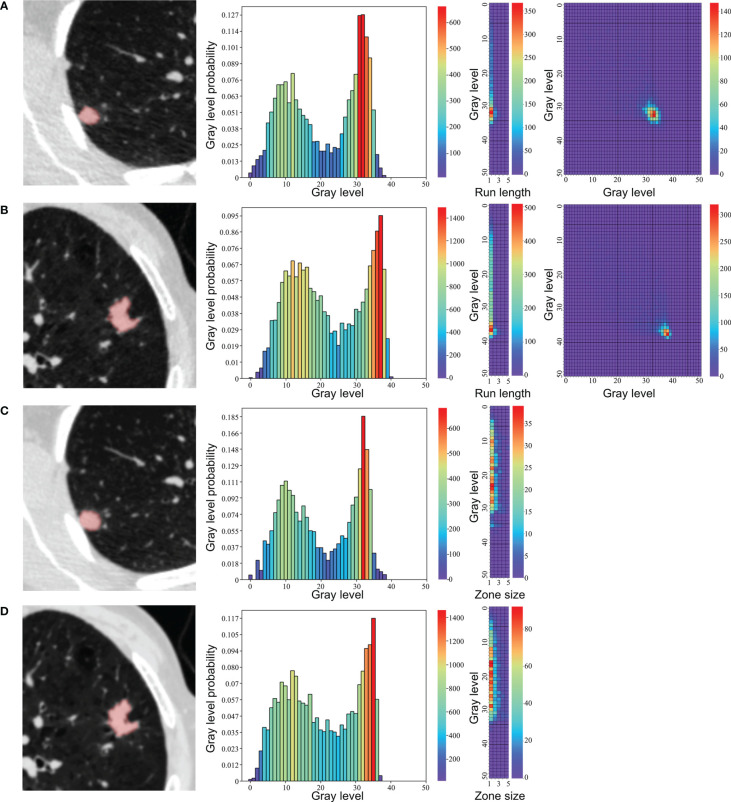
Representative segmentation results and texture feature maps of nodules. **(A, C)** A 43-year-old female with a granuloma, **(B, D)** A 75-year-old male with an adenocarcinoma. From left to right: **(A, B)** segmentation in low-dose CT, neighboring gray tone difference matrix, gray-level run length matrix, gray-level cooccurrence matrix. **(C, D)** segmentation in standard-dose CT, neighboring gray tone difference matrix, gray level size zone matrix.

All target nodules were automatically detected and segmented using uAI platform (United Imaging Healthcare, Shanghai, China), an artificial intelligence software basing on deep learning method (35, 36). No manual adjustments of the segmentation results were performed to avoid inter-observer and intra-observer variability. The representative segmentation results were shown in **Figure 1**. A total of 104 original radiomic features, including first order, shape, and texture features, were extracted from the target nodules using an open-source Python package (PyRadiomics, version 3.0, https://pyradiomics.readthedocs.io) (37). Further details of radiomic features are provided in the **Supplementary Material**.

### Quantifying Feature Reproducibility

The concordance correlation coefficient (CCC) was used to quantify reproducibility of extracted radiomic features between LDCT and standard-dose CT in the combined primary and validation cohorts (38). A radiomic feature with CCC ≥ 0.90 was then defined as a reproducible feature, as previously described (39–41). The percentage of features in each category with a CCC of ≥0.85, ≥0.90, and ≥0.95 was calculated, respectively.

### Feature Selection and Radiomic Signature Construction

Features were standardized using z-score normalization and then selected to build radiomic signature in the primary cohort of LDCT and standard-dose CT dataset respectively. Firstly, the Mann-Whitney U test was employed to select the features that were statistically different between groups (unadjusted *P* < 0.05), as the quantitative radiomic features did not have a normal distribution. Secondly, Spearman correlation analysis and minimum redundancy-maximum relevance (mRMR) (42) were sequentially conducted to exclude redundant radiomic features. Highly correlated features (Spearman correlation coefficient >0.9) were excluded and the top ranked 10 features were reserved. Thirdly, the least absolute shrinkage and selection operator (LASSO) method was used to select the most predictive features from the primary cohort (43). Finally, multivariate logistic regression with backward stepwise selection was applied to construct radiomic score (Rad-score), in which the stopping rule was the likelihood ratio test with Akaike’s information criterion (44). Rad-score of each patient in was calculated *via* a linear combination of the selected features and weighted by the respective coefficients.

### Radiomic Nomogram Construction

Independent factors for differentiating adenocarcinomas from benign nodules among Rad-score and clinical variables were identified by inputting significant variables found using univariate logistic regression analysis. Multivariable logistic regression analysis was applied to build radiomic nomogram for LDCT and standard-dose CT respectively, which was a visualized and individual tool that integrated independent factors to predict the probability of adenocarcinoma in the primary cohort.

### Performance of Radiomic Signature and Nomogram

The area under the curve (AUC) of receiver operating characteristic (ROC) curve was determined to evaluate the discrimination performance of the radiomic signature and radiomic nomogram of LDCT and standard-dose CT in both primary and validation cohorts. The sensitivity, specificity, accuracy, positive predictive value (PPV), and negative predictive value (NPV) were also calculated. To evaluate the calibration performance of radiomic nomogram, calibration curves were plotted. The Hosmer-Lemeshow (H-L) test was performed to assess the goodness-of-fit of radiomic nomogram.

### Clinical Utility

Decision curve analyses were conducted to estimate the clinical utility of the radiomic models and Lung-RADS by calculating the net benefits at a range of threshold probabilities in the combined primary and validation cohorts (45).

### Statistical Analysis

Statistical analysis was performed by R software (version 4.0.0, http://www.r-project.org), SPSS software (version 19.0, https://www.ibm.com), and MedCalc (v. 18.21, https://www.medcalc.org). The chi-squared test was used to compare the differences in gender, and group comparisons of age was performed using independent sample t-test. *P* < 0.05 was considered statistically significant.

The performance of Lung-RADS was also evaluated. The ROC of Lung-RADS was performed in both primary and validation cohorts according to that nodules of category 4A, 4B, and 4X were labeled as malignancy while category 2 and 3 as benign nodules. The corresponding sensitivity, specificity, accuracy, PPV, and NPV were calculated. Then comparisons of AUCs among radiomic models and Lung-RADS were performed using the Delong test in the primary and validation cohorts (46). In these pairwise models with significant difference in AUC, further comparisons of sensitivity and specificity were performed using the McNemar test in the combined cohort (47). Analyses were corrected for multiple comparisons using the false discovery rate (FDR) method (48).

## Results

### Clinical Characteristics

The baseline clinical-pathologic characteristics, including gender, age, Lung-RADS category, and histologic subtype of benign nodules in the primary and validation cohorts are listed in **Table 1**. There was no difference in gender between the adenocarcinoma group and the benign group in the primary or validation cohorts. Significant differences were found in age between the two groups in primary and validation cohorts (*P* = 0.003 and *P* < 0.0001, respectively).

**Table 1:** Characteristics of adenocarcinomas and benign nodules in the primary and validation cohorts.

Characteristic	Primary cohort (n = 98)			Validation cohort (n = 43)		
	Adenocarcinoma (n = 50)	Benign (n = 48)	*P*	Adenocarcinoma (n = 22)	Benign (n = 21)	*P*
Gender			0.69			0.55
Female	22	19		9	11	
Male	28	29		13	10	
Age (years)	61.32 ± 1.30	53.96 ± 1.44	0.0003	65.45 ± 1.70	53.33 ± 1.59	<0.0001
Lung-RADS category						
2, 3	2	27		0	14	
4A, 4B, 4X	48	21		22	7	
Histologic subtype						
Inflammation		17			10	
Granulomas		17			7	
Hamartoma		6			1	
PSP		5			0	
Other benign entities		3			3	

Age is shown in means ± standard deviation, and the other data are the number of nodules. Granulomas are caused by Mycobacterium tuberculosis, Cryptococcus neoformans, and other unspecified conditions. Other benign entities include intrapulmonary lymph node, fibroplasia, and lymphoid hyperplasia. Lung-RADS, Lung CT Screening Reporting and Data System; PSP, pulmonary sclerosing pneumocytoma.

### Feature Reproducibility

The reproducibility of radiomic features between LDCT and standard-dose CT regarding different feature categories is presented in the **Supplementary Material**. Shape and neighboring gray tone difference matrix (NGTDM) features were most reproducible (100%), followed by first order features (n = 16/18, 89%). Besides, 57% gray-level cooccurrence matrix (GLCM, n = 12/21), 50% gray level size zone matrix (GLSZM, n = 8/16), and 43% gray level dependence matrix (GLDM, n = 6/14) features were reproducible. Gray-level run length matrix (GLRLM) features were least reproducible (n = 5/16, 31%).

### Feature Selection and Radiomic Signature Construction

The process of feature selection is presented in the **Supplementary Material**. Finally, three features in LDCT (GLCM_DifferenceVariance, GLRLM_RunEntropy, and NGTDM_Strength) and two features in standard-dose CT (GLSZM_ZoneEntropy and NGTDM_Strength) were selected in the primary cohort. The representative maps of these texture feature were shown in **Figure 1**. The calculation formulas of Rad-score basing on these features with nonzero coefficients are presented in the **Supplementary Material**. Distributions of the Rad-score in the adenocarcinoma and benign groups in the primary and validation cohorts are shown in the **Supplementary Material**.

### Radiomic Nomogram Construction

**Figure 2:**
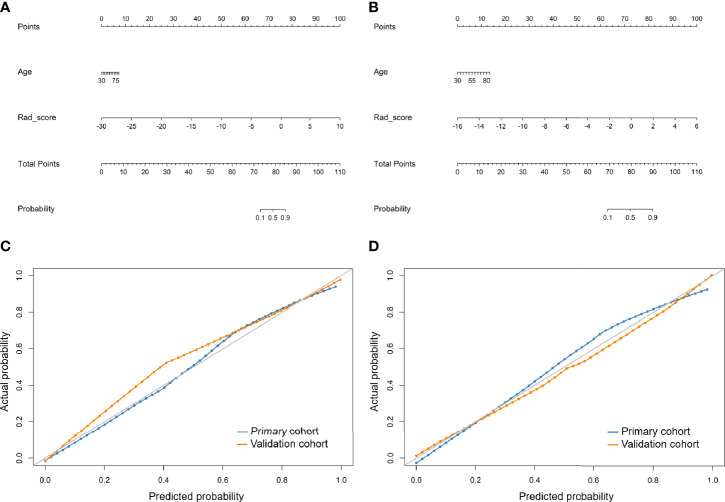
Developed radiomic nomograms and calibration curves for predicting the probability of adenocarcinoma. **(A)** Radiomic nomogram of low-dose CT. **(B)** Radiomic nomogram of standard-dose CT. **(C)** Calibration curve of radiomic nomogram of low-dose CT. **(D)** Calibration curve of radiomic nomogram of standard-dose CT. Rad_score, radiomic score.

According to univariate logistic regression analysis, age, Rad_score of LDCT, and Rad_score of standard-dose CT were significant independent differentiators of adenocarcinomas and benign nodules (**Table 2**), and they were integrated to develop the radiomic nomograms for predicting the probability of adenocarcinoma of LDCT and standard-dose CT respectively (**Figures 2A, B**). The calculation formulas of radiomic nomogram are presented in the **Supplementary Material**.

**Table 2:** Univariate and multivariate logistic regression analysis of factors for differentiating adenocarcinomas from benign nodules in the primary cohort.

Variables		Odds ratio	95% confidence interval	*P*
Univariate logistic regression				
Age		1.085	1.035–1.137	0.0008
Gender		1.199	0.537–2.680	0.658
Rad_score of low-dose CT		2.718	1.718–4.302	<0.0001
Rad_score of standard-dose CT		2.718	1.785–4.410	<0.0001
Multivariate logistic regression				
Low-dose CT	Rad_score	2.601	1.643–4.118	<0.0001
	Age	1.052	0.990–1.117	0.104
Standard-dose CT	Rad_score	2.638	1.713–4.061	0.088
	Age	1.053	0.992–1.118	<0.0001

Rad_score, radiomic score.

### Performance of Radiomic Signature, Radiomic Nomogram, and Lung-RADS

**Figure 3:**
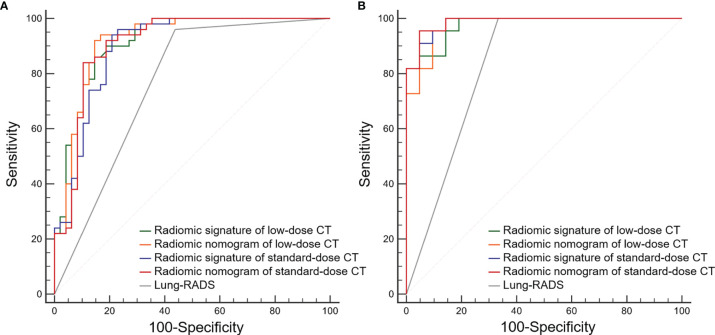
Receiver operating characteristic (ROC) curves of the radiomic models and Lung CT Screening Reporting and Data System (Lung-RADS) for differentiating adenocarcinomas from benign nodules. **(A)** Primary cohort. **(B)** Validation cohort.

The ROC curves of radiomic models and Lung-RADS are shown in **Figure 3**. The AUC, sensitivity, specificity, accuracy, PPV, and NPV of each model are shown in **Table 3**.

**Table 3:** Diagnostic performance the radiomic signature and radiomic nomogram of low-dose CT and standard-dose CT, and Lung-RADS for differentiating adenocarcinomas from benign nodules in the primary and validation cohorts.

	AUC (95% CI)	Sensitivity	Specificity	Accuracy	PPV	NPV
Primary cohort						
Radiomic signature of low-dose CT	0.915 (0.859–0.972)	0.860 (43/50)	0.854 (41/48)	0.857 (84/98)	0.860 (43/50)	0.854 (41/48)
Radiomic nomogram of low-dose CT	0.919 (0.861–0.977)	0.920 (46/50)	0.854 (41/48)	0.888 (87/98)	0.868 (46/53)	0.911 (41/45)
Radiomic signature of standard-dose CT	0.898 (0.833–0.963)	0.940 (47/50)	0.792 (38/48)	0.867 (85/98)	0.825 (47/57)	0.927 (38/41)
Radiomic nomogram of standard-dose CT	0.909 (0.946–0.972)	0.840 (42/50)	0.896 (43/48)	0.867 (85/98)	0.894 (42/47)	0.843 (43/51)
Lung-RADS	0.761 (0.685–0.837)	0.960 (48/50)	0.562 (27/48)	0.765 (75/98)	0.696 (48/69)	0.931 (27/29)
Validation cohort						
Radiomic signature of low-dose CT	0.976 (0.944–1.000)	0.864 (19/22)	0.857 (18/21)	0.860 (37/43)	0.864 (19/22)	0.857 (18/21)
Radiomic nomogram of low-dose CT	0.976 (0.941–1.000)	0.909 (20/22)	0.905 (19/21)	0.907 (39/43)	0.909 (20/22)	0.905 (19/21)
Radiomic signature of standard-dose CT	0.985 (0.960–1.000)	0.955 (21/22)	0.905 (19/21)	0.930 (40/43)	0.913 (21/23)	0.950 (19/20)
Radiomic nomogram of standard-dose CT	0.987 (0.965–1.000)	0.909 (20/22)	0.952 (20/21)	0.930 (40/43)	0.952 (20/21)	0.909 (20/22)
Lung-RADS	0.833 (0.730–0.937)	1.000 (22/22)	0.667 (14/21)	0.837 (36/43)	0.759 (22/29)	1.000 (14/14)

Unless otherwise specified, numbers in the parentheses were used to calculate percentages. Lung-RADS, Lung CT Screening Reporting and Data System; AUC, area under curve; CI, confidence interval; PPV, positive predictive value; NPV, negative predictive value.

The calibration curves of the radiomics nomogram of LDCT and standard-dose CT for the probability of adenocarcinoma demonstrated good agreement between prediction and observation in the primary and validation cohorts (**Figures 2C, D**). The H-L test yielded non-significant results in the both primary and validation cohorts of LDCT (*P* = 0.650 and 0.998) and standard-dose CT (*P* = 0.151 and 0.988), which suggested no departure from a perfect fit.

According to the DeLong test, the AUCs of the radiomic models were higher than that of Lung-RADS in the primary and validation cohorts (all correct *P* < 0.05), while there were no significant differences among the radiomic models (**Table 4**). The McNemar test results further showed the radiomic models had higher specificity than Lung-RADS in the combined cohort (all correct *P* < 0.05), while there were no significant differences of sensitivity between Lung-RADS and radiomic models (**Table 5**).

**Table 4:** Comparisons of area under the curves among the radiomic models and Lung-RADS in the primary and validation cohorts.

Pairwise comparison	Primary cohort		Validation cohort	
	*Z*	*P*	*Z*	*P*
Lung-RADS *vs.* Radiomic signature of low-dose CT	4.012	0.0001^*^	2.643	0.0082^*^
Lung-RADS *vs.* Radiomic nomogram of low-dose CT	4.123	<0.0001^*^	2.679	0.0074^*^
Lung-RADS *vs.* Radiomic signature of standard-dose CT	3.329	0.0009^*^	2.732	0.0063^*^
Lung-RADS *vs.* Radiomic nomogram of standard-dose CT	3.713	0.0002^*^	2.798	0.0051^*^
Radiomic signature of low-dose CT *vs.* Radiomic nomogram of low-dose CT	0.354	0.7236	<0.001	0.999
Radiomic signature of low-dose CT *vs.* Radiomic signature of standard-dose CT	1.058	0.2903	0.707	0.4794
Radiomic signature of low-dose CT *vs.* Radiomic nomogram of standard-dose CT	0.385	0.7003	0.928	0.3534
Radiomic nomogram of low-dose CT *vs.* Radiomic signature of standard-dose CT	1.037	0.2998	0.599	0.5492
Radiomic nomogram of low-dose CT *vs.* Radiomic nomogram of standard-dose CT	0.663	0.5075	0.841	0.4003
Radiomic signature of standard-dose CT *vs.* Radiomic nomogram of standard-dose CT	1.019	0.3084	0.402	0.6877

^*^Differences are significant at P < 0.05 corrected with false discovery rate. Lung-RADS, Lung CT Screening Reporting and Data System.

**Table 5:** Comparisons of sensitivity and specificity between Lung-RADS and radiomic models in the combined cohort.

Pairwise comparison	Sensitivity		Specificity	
	X^2^	*P*	X^2^	*P*
Lung-RADS *vs.* Radiomic signature of low-dose CT	4.900	0.0269	12.042	0.0005^*^
Lung-RADS *vs.* Radiomic nomogram of low-dose CT	0.167	0.6831	9.375	0.0022^*^
Lung-RADS *vs.* Radiomic signature of standard-dose CT	1.500	0.2207	14.087	0.0002^*^
Lung-RADS *vs.* Radiomic nomogram of standard-dose CT	4.900	0.0269	16.962	<0.0001^*^

^*^Differences are significant at P < 0.05 corrected with false discovery rate. Lung-RADS, Lung CT Screening Reporting and Data System.

### Clinical Utility

**Figure 4:**
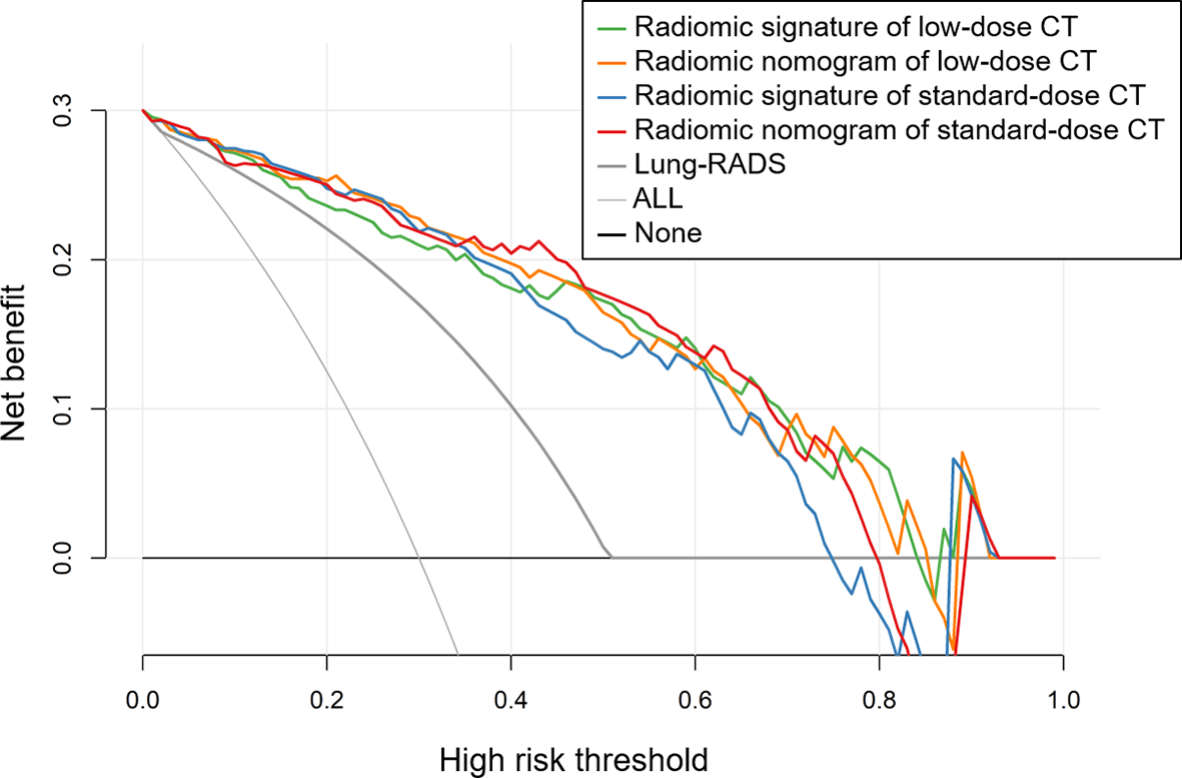
Decision curves of the radiomic models and Lung CT Screening Reporting and Data System (Lung-RADS). The decision curves showed that the model of radiomic signature of low-dose CT, radiomic nomogram of low-dose CT, radiomic signature of standard-dose CT, and radiomic nomogram of standard-dose CT added more net benefit than Lung-RADS in differentiating adenocarcinomas from benign nodules within the range of the threshold probability of 0.02 to 0.84, 0.02 to 0.85, 0.02 to 0.74, and 0.02 to 0.79, respectively.

The results of decision curve analyses for the radiomic models and Lung-RADS are presented in **Figure 4**. The decision curves showed that the model of radiomic signature of low-dose CT, radiomic nomogram of low-dose CT, radiomic signature of standard-dose CT, and radiomic nomogram of standard-dose CT added more net benefit than Lung-RADS in differentiating adenocarcinomas from benign nodules within the range of the threshold probability of 0.02 to 0.84, 0.02 to 0.85, 0.02 to 0.74, and 0.02 to 0.79, respectively.

## Discussion

In the present study, we investigated the ability of radiomic models based on LDCT and standard-dose CT to distinguish adenocarcinomas from benign lesions in patients with solid solitary pulmonary nodules. We found the AUCs of LDCT-based radiomic models were equivalent to that of standard-dose CT. All the radiomic models showed higher specificity than the Lung-RADS approach, which was consistent with previous report (12, 17). We also assessed the reproducibility of radiomic features of solid solitary pulmonary nodules between LDCT and standard-dose CT examinations *in vivo*. Generally, shape and first order features were more reproducible than texture features except for NGTDM features.

Radiologists usually stratify pulmonary nodules in lung cancer screening by interpreting characteristics such as location, attenuation, diameter, volume, and margin. The differential diagnosis of solitary solid nodules may be more difficult than that of sub-solid nodules. More than 90% of pathologically confirmed sub-solid nodules were malignant in China (49), while the malignancy rate of solid nodules was 66.6% in previous study (50) and 51.1% in our study. Several possible reasons may lead to the overtreatment of solid nodules in China. First, with high tuberculosis incidence in this country, indeterminate solid nodules caused by granulomas or other inflammations were usually larger than 8 mm with irregular shape and border. These benign nodules had overlapping characteristics with adenocarcinomas in location, size, and morphology (51–53). Thus, the Lung-RADS categorization of these benign nodules were equal to or beyond 4A, resulting in low specificity. The radiomic models developed in this study had higher specificity and lower false-positive rate in distinguishing adenocarcinomas from benign lesions than the Lung-RADS, and they might help clinicians avoid choosing too aggressive approach. Second, 41 of 69 benign nodules in this study were categorized as Lung-RADS 2 or 3, and they were all pathologically confirmed by surgery. The result indicated the great fear of missing malignant nodules for both patients and surgeons in China, especially in the cancer hospital. Because the medical environment of China tends to favor cautiousness from both patients and clinicians (54).

This study also addressed a very important question that radiomic models based on LDCT and standard-dose CT had equivalent diagnostic performance to differentiate adenocarcinomas from benign lesions in solid nodules. NGTDM_Strength was highly reproducible and thus the common significant texture feature related to benign nodules in both LDCT and standard-dose CT models. Higher value of NGTDM_Strength indicates an image with slower change in intensity but larger coarse differences in gray level intensities. It suggested benign nodules were more homogeneous than adenocarcinomas. Besides, the other significant texture features related to adenocarcinomas included GLCM_DifferenceVariance and GLRLM_RunEntropy in LDCT model and GLSZM_ZoneEntropy in standard-dose CT model. They all indicated that adenocarcinomas had more heterogeneity than benign nodules in the texture patterns. The shape and first order features, representing the morphology and attenuation characteristics, were highly reproducible as radiation dose changed. However, they were not included in LDCT or standard-dose CT models, suggesting the deficiency of traditional image features to stratify the indeterminate solid nodules.

We acknowledged several limitations in this study. First, this was a single-center retrospective study with relatively small sample size. The advantage was the standardization of acquisition parameters, avoiding potential confounding variability caused by heterogeneous parameters and image preprocessing (21, 25). Further multi-center study with larger datasets is needed to validate the reported radiomic models. Second, only pathologically confirmed nodules were enrolled and nodules with undefined border resulting in poor segmentation were excluded, leading to potential selection bias. Third, nodule segmentation was performed with an artificial intelligence software basing on deep learning method and the underlying parameters were inherently in black box. The advantage of automatic segmentation method was high reproducibility, avoiding inter-observer and intra-observer variability that resulting from manual segmentation.

In conclusion, the diagnostic performance of radiomic models based on LDCT and standard-dose CT to differentiate adenocarcinomas from benign lesions in solid pulmonary nodules were equivalent. These radiomic models had higher specificity and lower false-positive rate than Lung-RADS. The LDCT-based radiomic model might be an effective tool for reducing overdiagnosis and overtreatment of solid pulmonary nodules in lung cancer screening.

## Data Availability Statement

The original contributions presented in the study are included in the article/**Supplementary Material**. Further inquiries can be directed to the corresponding author.

## Ethics Statement

The studies involving human participants were reviewed and approved by the Ethics Review Board of Sichuan Cancer Hospital & Institute, School of Medicine, University of Electronic Science and Technology of China. Written informed consent for participation was not required for this study in accordance with the national legislation and the institutional requirements.

## Author Contributions

JL, JR, and PZ conceived and designed the study. HQ, YL, XY, and CH collected the data. JL and HX analyzed the data and drafted the manuscript. All authors reviewed the manuscript and PZ revised the final manuscript. HQ and PZ provided funding for the study. All authors contributed to the article and approved the submitted version.

## Funding

This study was supported by Sichuan Science and Technology Program (grant numbers 2019YJ0585, 2021YFS0075) and Chengdu Science and Technology Program (grant number 2018-YF05-01134-SN).

## Conflict of Interest

The authors declare that the research was conducted in the absence of any commercial or financial relationships that could be construed as a potential conflict of interest.
