# Supplementary material for: Comparison of Radiomic Models Based on Low-Dose and Standard-Dose CT for Prediction of Adenocarcinomas and Benign Lesions in Solid Pulmonary Nodules
Source: Front Oncol. 2021 Feb 2;10:634298. doi: 10.3389/fonc.2020.634298 (PMC7884759; doi:10.3389/fonc.2020.634298)
Supplement: Supplementary file 1 [file DataSheet_1.zip › Supplementary Material.docx]

***Supplementary Material***

**Supplementary Data**

**Calculation formulas of radiomic score**

Rad_score of low-dose CT = -1.410382 -1.260785 × GLCM_DifferenceVariance + 1.222757 × GLRLM_RunEntropy -4.210459 × NGTDM_Strength

GLCM_DifferenceVariance, mean = 16.876092, standard deviation = 5.498584.

GLRLM_RunEntropy, mean = 5.239541, standard deviation = 0.213623.

NGTDM_Strength, mean = 1.947357, standard deviation = 1.877827.

Rad_score of standard-dose CT = -0.6908825 + 1.46795 × GLSZM_ZoneEntropy -2.522378 × NGTDM_Strength

GLSZM_ZoneEntropy, mean = 6.578316, standard deviation =0.393412.

NGTDM_Strength, mean = 1.92269, standard deviation = 1.716712.

**Calculation formulas of radiomic nomogram**

Rad_nomo of low-dose CT = -2.920337 + 0.050297 × Age + 0.955870 × Rad_score

Rad_nomo of standard-dose CT = -3.005581 + 0.051814 × Age + 0.970000 × Rad_score

*Values of radiomic features were standardized with Z-scores according to the formula: standardized value = (raw value - mean)/standard deviation. Rad_score, radiomic score; Rad_nomo, radiomic nomogram; GLCM, gray-level cooccurrence matrix; NGTDM, neighbouring gray tone difference matrix; GLSZM, gray level size zone matrix.*

**Supplementary Table.** Overview of all radiomic features.

| **Category** | **Features** | **Counts** |
| --- | --- | --- |
| Shape | Elongation, Flatness, Least axis length, Major axis length, Maximum 2D diameter (column), Maximum 2D diameter (row), Maximum 2D diameter (slice), Maximum 3D diameter, Mesh volume, Minor axis length, Sphericity, Surface area, Surface area to volume ratio, Voxel volume | 14 |
| First order | 10 Percentile, 90 Percentile, Energy, Entropy, Interquartile range, Kurtosis, Maximum, Mean absolute deviation, Mean, Median, Minimum, Range, Robust mean absolute deviation, Root mean squared, Skewness, Total energy, Uniformity, Variance | 18 |
| Texture |  |  |
| GLCM | Autocorrelation, Cluster prominence, Cluster shade, Cluster tendency, Contrast, Correlation, Difference average, Difference entropy, Difference variance, Inverse difference normalized (Idn), Inverse difference (Id), Inverse difference moment normalized (Idmn), Inverse difference moment (Idm), Informational measure of correlation 1 (Imc1), Informational measure of correlation 2 (Imc2), Inverse variance, Joint average, Joint energy, Joint entropy, Maximum probability, Sum entropy | 21 |
| GLRLM | Gray level non-uniformity (GLN), Gray level non-uniformity normalized (GLNN), Gray level variance (GLV), High gray level run emphasis (HGLRE), Long run emphasis (LRE), Long run high gray level emphasis (LRHGLE), Long run low gray level emphasis (LRLGLE), Low gray level run emphasis (LGLRE), Run entropy (RE), Run length non-uniformity (RLN), Run length non-uniformity normalized (RLNN), Run percentage (RP), Run variance (RV), Short run emphasis (SRE), Short run high gray level emphasis (SRHGLE), Short run low gray level emphasis (SRLGLE) | 16 |
| GLSZM | Gray level non-uniformity (GLN), Gray level non-uniformity normalized (GLNN), Gray level variance (GLV), High gray level zone emphasis (HGLZE), Large area emphasis (LAE), Large area high gray level emphasis (LAHGLE), Large area low gray level emphasis (LALGLE), Low gray level zone emphasis (LGLZE), Size zone non-uniformity (SZN), Size zone non-uniformity normalized (SZNN), Small area emphasis (SAE), Small area high gray level emphasis (SAHGLE), Small area low gray level emphasis (SALGLE), Zone entropy (ZE), Zone percentage (ZP), Zone variance (ZV) | 16 |
| GLDM | Dependence entropy (DE), Dependence non-uniformity (DN), Dependence non-uniformity normalized (DNN), Dependence variance (DV), Gray level non-uniformity (GLN), Gray level variance (GLV), High gray level emphasis (HGLE), Large dependence emphasis (LDE), Large dependence high gray level emphasis (LDHGLE), Large dependence low gray level emphasis (LDLGLE), Low gray level emphasis (LGLE), Small dependence emphasis (SDE), Small dependence high gray level emphasis (SDHGLE), Small dependence low gray level emphasis (SDLGLE) | 14 |
| NGTDM | Busyness, Coarseness, Complexity, Contrast, Strength | 5 |

*GLCM, gray-level cooccurrence matrix; GLRLM, gray-level run length matrix; GLSZM, gray level size zone matrix; GLDM, gray level dependence matrix; NGTDM, neighbouring gray tone difference matrix.*

**Supplementary Figures**

**Supplementary Figure 1.** Percentage of reproducible features between low-dose and standard-dose CT for different feature categories. Reproducibility is shown for 3 different cutoffs for CCC (≥ 0.85, ≥ 0.90, and ≥ 0.95). CCC, concordance correlation coefficient; GLCM, gray-level cooccurrence matrix;, GLRLM, gray-level run length matrix; GLSZM, gray level size zone matrix; GLDM, gray level dependence matrix; NGTDM, neighbouring gray tone difference matrix.

**Supplementary Figure 2.** Feature selection process. **(A)** Feature selection workflow for low-dose CT and standard-dose CT. **(B-E)** Feature selection using the LASSO binary logistic regression model for low-dose CT **(B, C)** and standard-dose CT **(D, E)**. **(B, D)** Tuning parameter lambda (λ) is chosen in the LASSO model using 10-fold cross-validation via minimum criteria (1-SE criteria). Binomial deviances from the LASSO regression cross-validation procedure are plotted as a function of log (λ). **(C, E)** LASSO coefficient profiles of the features show vertical lines that are drawn at the value selected using 10-fold cross-validation, and optimal λ results in 6 nonzero coefficients in both low-dose CT and standard-dose CT datasets. LASSO, least absolute shrinkage and selection operator; mRMR, minimum redundancy-maximum relevance.

**Supplementary Figure 3.** Distributions of Rad-score in the adenocarcinoma and benign groups in the primary and validation cohorts. **(A)** Primary cohort of low-dose CT. **(B)** Validation cohort of low-dose CT. **(C)** Primary cohort of standard-dose CT. **(D)** Validation cohort of standard-dose CT. Rad_score, radiomic score; M, adenocarcinomas; B, benign nodules.
