# Supplementary figures and images for: Comparison of Radiomic Models Based on Low-Dose and Standard-Dose CT for Prediction of Adenocarcinomas and Benign Lesions in Solid Pulmonary Nodules
Source: Front Oncol. 2021 Feb 2;10:634298. doi: 10.3389/fonc.2020.634298 (PMC7884759; doi:10.3389/fonc.2020.634298)

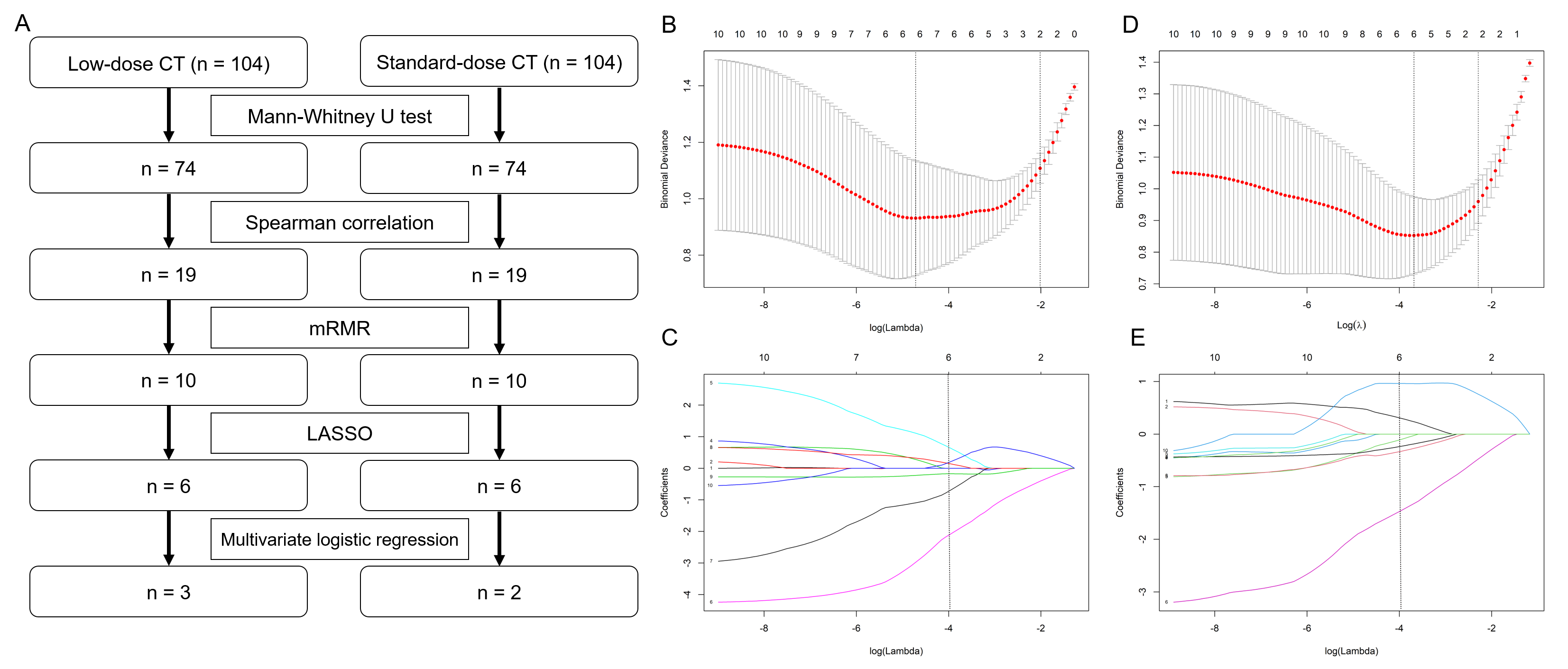

Supplement: Supplementary file 1 [file DataSheet_1.zip › Supplementary Figure 2.tif]

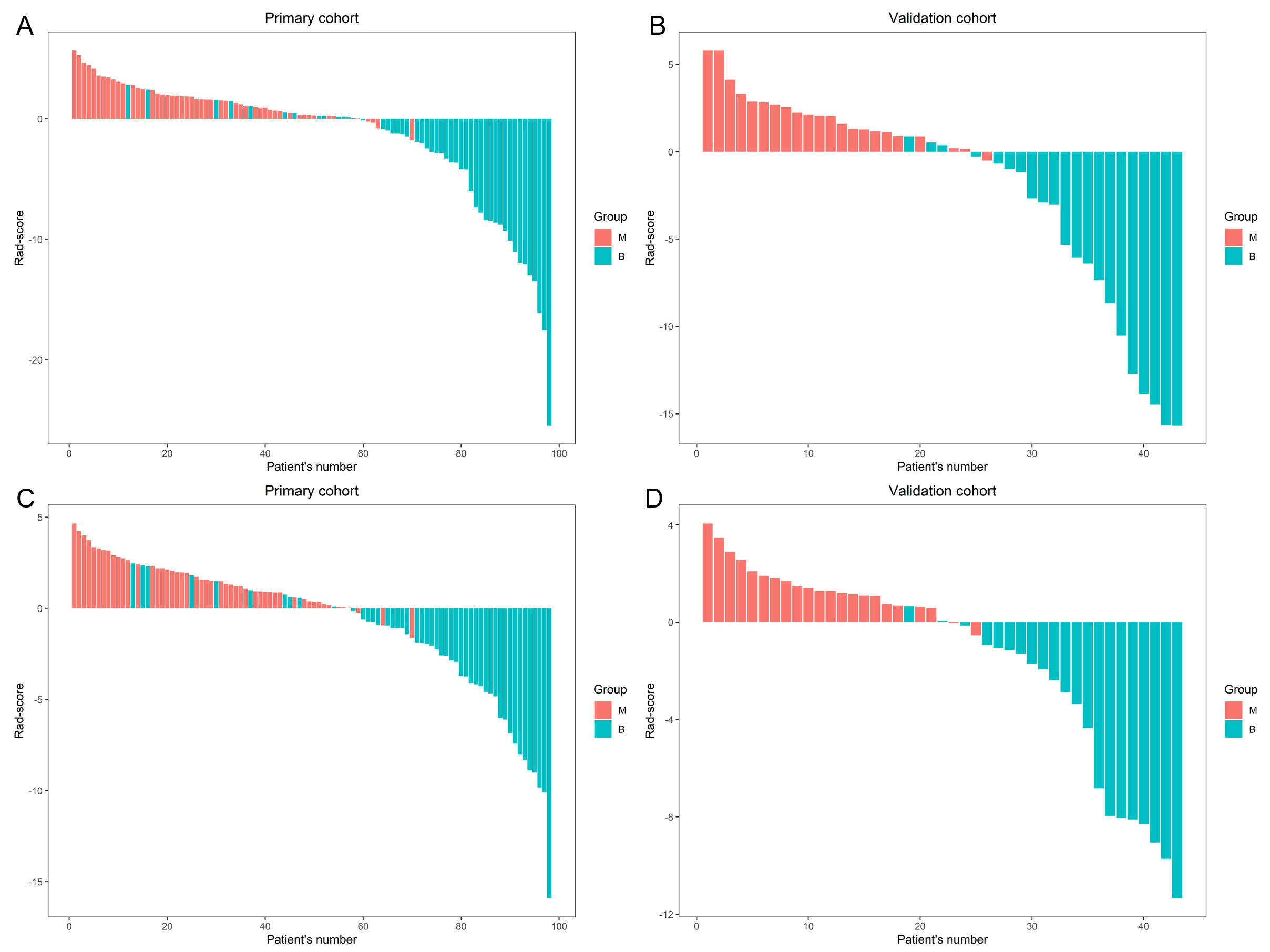

Supplement: Supplementary file 1 [file DataSheet_1.zip › Supplementary Figure 3.tif]

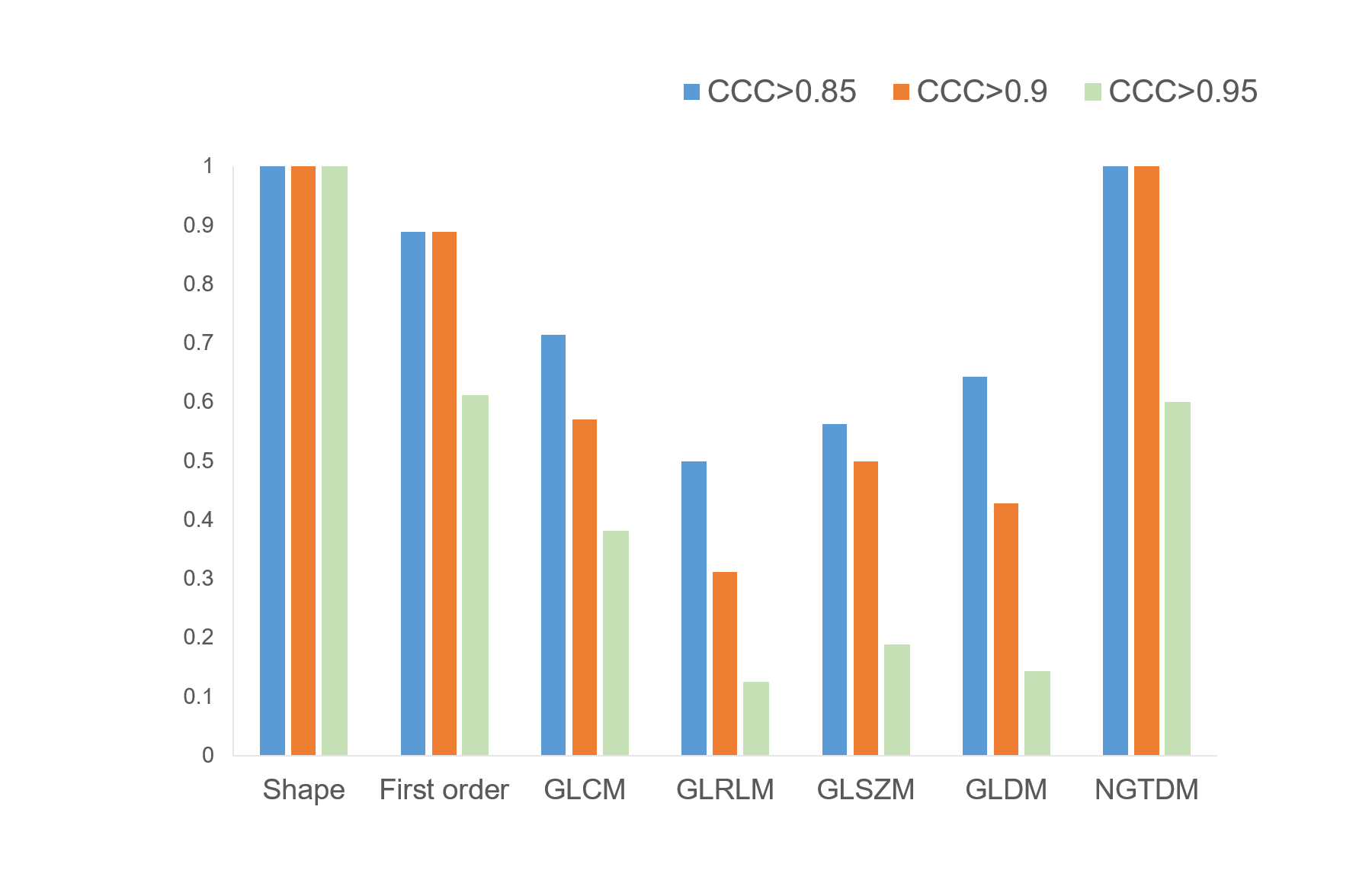

Supplement: Supplementary file 1 [file DataSheet_1.zip › Supplementary Figure 1.tif]
